# Therapeutic efficacy of pentoxifylline on proteinuria and renal progression: an update

**DOI:** 10.1186/s12929-017-0390-4

**Published:** 2017-11-13

**Authors:** Yung-Ming Chen, Wen-Chih Chiang, Shuei-Liong Lin, Tun-Jun Tsai

**Affiliations:** 1 Renal Division, Department of Medicine, National Taiwan University Hospital, College of Medicine, National Taiwan University, No. 7, Chung-Shan South Road, Taipei, Taiwan; 2 Graduate Institute of Physiology, College of Medicine, National Taiwan University, No. 1, Jen-Ai Road, Section 1, Taipei, Taiwan

**Keywords:** CKD, ESRD, Pentoxifylline, Proteinuria, Renin-angiotensin system

## Abstract

Blood pressure control with renin-angiotensin system (RAS) blockade has remained the gold standard for treating patients with proteinuric chronic kidney disease (CKD) up to date. Nevertheless, RAS blockade slows but does not halt the progression of kidney disease, thus highlighting the need to search for additional therapeutic approaches. The nonselective phosphodiesterase (PDE) inhibitor pentoxifylline (PTX) is an old drug that exhibits prominent anti-inflammatory, anti-proliferative and anti-fibrotic activities both in vitro and in vivo. Studies in human subjects have shown that PTX monotherapy decreases urinary protein excretion, and add-on therapy of PTX to background RAS blockade additively reduces proteinuria in patients with CKD of various etiology. More recent studies find that PTX combined with RAS blockade delays the decline of glomerular filtration rate in diabetic patients with mild to moderate CKD, and reduces the risk of end-stage renal disease in diabetic and non-diabetic patients in late stage of CKD with high proteinuria levels. In this review, we update the clinical trial results of PTX as monotherapy, or in conjunction or in comparison with RAS blockade on patients with proteinuria and CKD, and propose a mechanistic scheme explaining the renoprotective activities of this drug.

## Background

Current practice guidelines recommend blood pressure control with inhibitors of the renin-angiotensin system (RAS) as the gold standard therapy for patients with proteinuric chronic kidney disease (CKD) [1–3]. Blocking the RAS by either angiotensin converting enzyme inhibitors (ACEIs) or angiotensin receptor blockers (ARBs) slows but does not stop the progression of kidney disease [4]. This highlights the need to search for additional therapeutic approaches beyond RAS blockade [5]. However, new drug development for kidney diseases has been limited, and except for some glucose-lowering drugs [6], no novel agents targeting renal progression have been marketed since the new millennium [7, 8]. Recently, the cyclic nucleotide phosphodiesterase (PDE) has emerged as a promising target for pharmacological intervention against CKD progression [9, 10]. The nonselective PDE inhibitor pentoxifylline (PTX) is an old drug that exhibits prominent anti-inflammatory, anti-proliferative and anti-fibrotic activities both in vitro and in vivo [11, 12]. In addition to its classic indication for intermittent claudication [13–15], PTX has been used off-label to treat a variety of inflammatory and/or fibrotic disorders, including that arise from the kidney [16–21]. To understand further the impact of PTX therapy on renal diseases since our previous review [22, 23], we conducted a search of the literature using the PubMed, EMBASE, Cochrane Central Register of Controlled Trials, and Cochrane Database of Systematic Reviews. Key words used as search terms were “pentoxifylline”, “diabetic proteinuria”, “non-diabetic proteinuria”, and “renal progression”, “chronic kidney disease”, “meta-analysis”, and “systematic review”. Results were limited to studies in human subjects published in the English language after December 2004, and those studies using PTX with or without RAS blockade. Studies focused on the effects of PTX on drug induced nephrotoxicity were excluded. Using the search methods described above, 27 relevant articles were retrieved and evaluated. The populations studied were patients with CKD across all 5 stages. We stratify these studies based on the treatment intervals and primary endpoints, i.e., short-term effects (≦6 months), either monotherapy or add-on existing RAS blockade, on proteinuria; longer term (≧12 months) add-on therapy to background RAS blockers on renal function. Of the 27 studies, 4 analyzed the effect of PTX on non-diabetic proteinuria, 13 assessed the effect of PTX on diabetic proteinuria, including 3 meta-analyses and systematic reviews, and 10 evaluated the changes in estimated glomerular filtration rate (eGFR), including 4 randomized clinical trials, 3 meta-analyses and systematic reviews and 3 cohort observational studies. In this narrative review, we update the clinical trial results of PTX as monotherapy, or in conjunction or in comparison with RAS blockade, on patients with proteinuria and CKD, and propose a mechanistic scheme based on our prior works to account for the renoprotective activities of this drug.

## Main text

### Efficacy of PTX on non-diabetic proteinuria

Table 1 shows representative studies examining the efficacy of PTX on non-diabetic patients with proteinuria. Chen et al. [24]. reported that treatment with PTX at a dose of 800 mg daily for 6 months decreased proteinuria from averaging 2.82 to 1.79 g/g Creatinine (g/gCr) in 17 primary glomerulonephritic patients. The efficacy was associated with reduced urinary monocyte chemoattractant protein (MCP)-1 excretion, proposing a mechanistic basis for PTX in non-diabetic patients with proteinuria. Then, in a placebo-controlled, cross-over study, Renke et al. [25] found PTX at a dose of 1200 mg daily reduced proteinuria by 26% in comparison with placebo in 22 non-diabetic patients with proteinuria between 0.4 and 4.3 g/day and eGFR >30 mL/min/1.73 m^2^. No differences were found between crossover periods in C-reactive protein (CRP), α1-microglobulin, and urinary N-acetyl-beta-d-glucosaminidase. More recently, in a double-blind, placebo-controlled trial, Badri et al. [26] observed add-on PTX to background RAS blockade at a dose of 800–1200 mg daily for 6 months additively decreased proteinuria in 18 patients with membranous nephropathy displaying urinary protein excretion >500 mg/day.

**Table 1:** Studies examining the efficacy of PTX on non-diabetic proteinuria

Investigators, years [Ref.]	Patients, number	Study design	PTX dose, duration	Background RAS blockade	Main outcome findings	Safety profiles
Chen et al., 2006 [24]	Subnephrotic GN CKD stages 1 to 4, *N* = 17	Open-label, single arm	800 mg/day (400 mg twice daily), 6 months	No RAS blockade or immune-suppressive agents	The use of PTX reduced proteinuria, in conjunction with a decrease in urinary MCP-1.	None discontinued the treatment due to adverse effects. One (6%) patient experienced gastric upset that disappeared after taking the drug after meals.
Shu et al., 2007 [27]	Chronic allograft nephropathy with proteinuria 2.65 ± 2.15 g/day and mean eGFR 38 mL/min (serum creatinine < 3 mg/dL), N = 17	Open-label, single arm	1200 mg/day (400 mg three times daily), 6 months	No RAS blockade, but triple immune- suppressive agents (corticosteroid, calcineurin inhibitor, mycophenolate mofetil)	PTX resulted in temporary reduction of proteinuria, and CD4+ cells bearing TNF-α and IL-10. More than 50% patients displayed stable graft function at end of 6 months	Four (23.5%) patients reported adverse effects. One patient discontinued the treatment due to headache, two others experienced transient dizziness and remained in the study. One female developed menorrhagia which resolved after withholding PTX during menstrual periods.
Renke et al., 2010 [25]	Non-diabetic CKD stages 1 to 3 (eGFR 37–178 mL/min) with proteinuria (0.4–4.3 g/day), *N* = 22	Randomized, placebo controlled cross-over Placebo ➔ PTX PTX ➔ placebo (8 dropped-out)	1200 mg/day, 8 weeks	ACEI and/or ARB, with 14 (64%) patients receiving combined ACEI and ARB treatment	PTX reduced proteinuria (by 26%) compared to placebo. No differences in hsCRP, α1-microglobulin, urine NAG, 15-F(2)t-isoprostane.	Five patients (23%) taking PTX withdrew from the study due to digestive symptoms (nausea, dyspepsia, diarrhea). Another 3 patients resigned from participation due to personal reasons.
Badri et al., 2013 [26]	Membranous nephropathy with proteinuria > 0.5 g/day, CKD stages 3 to 4, *N* = 18	Randomized, double-blind, placebo controlled PTX + RAS blockade (*n* = 12) Placebo + RAS blockade (*n* = 6)	800–1200 mg/day, 6 months	ACEI and/or ARB, Immune-suppressive agents in 5 (28%) patients	PTX reduced proteinuria without affecting eGFR	PTX therapy was well tolerated in this study. Two (11%) patients experienced nausea that disappeared after taking the drug after meals. No patient discontinued the drug because of adverse effects.

*ACEI* angiotensin converting enzyme inhibitor, *ARB* angiotensin receptor blocker, *CKD* chronic kidney disease, *eGFR* estimated glomerular filtration rate, *Ref* reference, *GN* glomerulonephritis, *hsCRP* high sensitivity C-reactive protein, *IL* interleukin, *MCP*-1 monocyte chemoattractant protein-1, *NAG* N-acetyl-beta-D-glucosaminidase, *PTX* pentoxifylline, *RAS* renin-angiotensin system, *TNF*-α, tumor necrosis factor-α

In addition to native kidney disease, PTX also works in the setting of transplant kidneys. An early randomized double-blind trial found that PTX at a dose of 800–1200 mg daily ameliorated the consequences of rejection on graft survival comparing to placebo during the first 6 months after transplantation [27]. Subsequent studies revealed temporary anti-proteinuric effects of PTX in biopsy proved chronic allograft nephropathy under RAS blockade and triple immunosuppressive therapy. The graft function was stabilized in more than half of PTX-treated patients at the end of 6-month follow-up, supporting a renoprotective role of this drug for graft disease [28, 29]. Unfortunately, all the studies mentioned above suffered the caveats of small sample size, short periods of observation and bias-prone designs, which preclude drawing a firm conclusion for the efficacy of PTX on non-diabetic patients with proteinuria.

### Efficacy of PTX on diabetic proteinuria

Diabetic kidney disease (DKD) or diabetic nephropathy has become the single most important primary etiology of end-stage renal disease (ESRD) worldwide [30], and the pathophysiology of which has been attributed to various metabolic and hemodynamic factors [31, 32]. Not surprisingly, glycemic control and blood pressure control with RAS blockade are widely accepted as the standard therapy for people with DKD. Besides these conventional approaches, inflammatory processes induced by tumor necrosis factor (TNF)-α has emerged as an alternative therapeutic target for patients with DKD [33–35]. Consistent with this notion, PTX which is a known inhibitor for TNF-α [36, 37] has been used as an anti-proteinuric agent in DKD patients [38]. Table 2 lists the representative studies assessing the efficacy of PTX on proteinuria in DKD patients. First, Aminorroaya et al. [39] and Rodríguez-Morán et al. [40] observed PTX at a dose of 400 mg three times daily displayed anti-proteinuric effects comparable to that achieved with captopril 25 mg three time daily in non-hypertensive patients with type 2 diabetes. These studies showed that PTX is non-inferior to ACEI for the anti-proteinuric effect, suggesting a role of PTX in the management of diabetic proteinuria. Then, in a randomized, open-label trial, Navarro et al. [41] found add-on PTX at a dose of 1200 mg daily to background ARB for 4 months additively decreased proteinuria in patients with type 2 diabetes. This extra anti-proteinuric effect of PTX was associated with reduced serum and urinary levels of TNF-α. However, only the change of urinary TNF-α correlated with the change of albuminuria. Meanwhile, in another trial using double-blind, placebo-controlled design, PTX at a dose of 1200 mg daily for 4 months reduced both high (glomerular) and low (tubular) molecular weight urinary protein excretion in normotensive type 2 diabetes patients with microalbuminuria [42].

**Table 2:** Studies examining the efficacy of PTX on diabetic proteinuria

Investigators, years [Ref.]	Patients, number	Study design	PTX dose, duration	Background RAS blockade	Main outcome findings	Safety profiles
Original investigation						
Aminorroaya et al., 2005 [39]	Hypertensive type 2 diabetes with proteinuria >300 mg/day, CKD stages 1 to 3, *N* = 40	Randomized, open-label, crossover PTX ➔captopril (*n* = 20) Captopril ➔ PTX (*n* = 19) 1 dropped-out	1200 mg/day (400 mg three times daily), 8 weeks	Captopril 25 mg thrice a day	Both PTX and captopril reduced macroalbuminuria. (PTX: 1.4 to 1.0 g/day; Captopril: 1.3 to 0.8 g/day)	PTX and captopril treatment was well tolerated, although 1 (5%) patient on the captopril arm withdrew due to the development of dry cough.
Rodriguez-Morán et al., 2005 [40]	Normotensive type 2 diabetes with microalbuminuria, *N* = 130	Randomized, open-label, equivalent PTX (*n* = 65) 1 dropped-out Captopril (*n* = 65) 3 dropped-out	1200 mg/day (400 mg three times daily), 6 months	Captopril 25 mg thrice a day	Both PTX and captopril reduced microalbuminuria. (PTX: 101.1 to 23.1 μg/min; Captopril: 102.0 to 23.9 μg/min)	One (1.5%) patient in the PTX group and 3 (5%) patients in the captopril group withdrew from the study due to headache and dry cough, respectively.
Navarro et al., 2005 [41]	Normotensive type 2 diabetes, persistent albuminuria >400 mg/day, CKD stage 1, *N* = 61	Randomized, open-label controlled PTX plus ARB (*n* = 30) ARB (*n* = 31)	1200 mg/day (600 mg twice daily), 4 months	Recommended dose of ARB	Add-on PTX reduced albuminuria (900 to 791 mg/day), whereas ARB did not (910 to 900 mg/day). Add-on PTX decreased serum and urinary levels of TNF-α, but only the change of urinary TNF-α correlated with the change of albuminuria.	Four (13%) patients developed dizziness, and 3 (10%) patients complained of dyspepsia in the PTX group. These were all transient, and no patient withdrew from the study as a result of PTX adverse effects.
Rodriguez-Morán et al., 2006 [42]	Normotensive type 2 diabetes with microalbuminuria, N = 40	Randomized, double-blind, placebo controlled PTX (n = 20) Placebo (*n* = 20)	1200 mg/day (400 mg three times daily), 4 months	No RAS blockade	PTX reduced urinary excretion of both high- & low- molecular weight proteins in comparison with the placebo.	No subjects dropped out, nor were there serious adverse events or side effects. Four (13%) patients receiving PTX experienced mild headache in the first month that did not require treatment.
Roozbeh et al., 2010 [45]	Type 2 diabetes with overt proteinuria (> 500 mg/day), CKD stage 1, *N* = 74	Randomized, open-label controlled Captopril (n = 37) 2 dropped-out PTX + captopril (*n* = 37) 2 dropped-out	1200 mg/day (400 mg three times daily), 6 months	Captopril (25 mg thrice a day)	PTX plus captopril led to greater reduction in proteinuria than captopril group. (PTX: 2.9 to 1.3 g/day; Captopril: 2.8 to 2.0 g/day)	One (3%) patient receiving PTX withdrew from the study due to nausea.
Oliaei et al., 2011 [43]	Type 2 diabetes with UPCR >500 mg/day; CKD stages 1 to 2, *N* = 56	Randomized, double-blind, placebo controlled PTX (n = 28) Placebo (*n* = 28)	1200 mg/day (400 mg three times daily), 3 months	ACEI and/or ARB	The use of PTX led to reduction of proteinuria, compared to the placebo group	No adverse effects or intolerance to drug were found during the period of treatment.
Ghorbani et al., 2012 [44]	Type 2 diabetes with persistent proteinuria >150 mg/day; CKD stages 1 to 3, *N* = 100	Randomized, double-blind, controlled PTX plus ACEI + ARB (n = 50), 6 dropped-out ACEI + ARB (*n* = 50)	400 mg/day, 6 months	ACEI (enalapril) plus ARB (losartan)	Add-on PTX additively reduced proteinuria after 3 months, independently of BP or metabolic control	In the PTX group, 1 (2%) patient with chest pain and dyspnea, 1 (2%) patient with retinal hemorrhage and 4 (8%) patients with intractable nausea and vomiting withdrew from the study.
Han et al., 2015 [46]	Type 2 diabetes with CKD stages 1 to 3, *N* = 174	Randomized double-blind, placebo controlled PTX + ARB (*n* = 52) 35 dropped-out Placebo + ARB (*n* = 70) 17 dropped-out	1200 mg/day (400 mg three times daily), 6 months	ARB	By using per protocol analysis, add-on PTX reduced proteinuria and improved glucose control and insulin resistance without decreasing serum TNF-α levels.	The frequency of adverse effects (dyspepsia, nausea, vomiting, gastric reflux, diarrhea and headache).was higher in the PTX group. Thirteen (15%) patients in the PTX group and 5 (6.5%) patients in placebo group withdrew from the study due to adverse effects.
Shahidi et al., 2015 [49]	Type 2 diabetes with microalbuminuria CKD stages 1 to 2, *N* = 50	Randomized double-blind, placebo-controlled PTX + usual care (n = 25), 5 dropped-out Placebo + usual care (*n* = 25), 5 dropped-out	1200 mg/day (400 mg three times daily), 6 months	Usual care with ACEI and/or ARB plus protein intake <0.8 g/kg/day and HbA1c < 8%	PTX plus usual care failed to reduce albuminuria compared with placebo plus usual care.	Ten patients (5 (20%) patients each in placebo and PTX groups) withdrew from the study due to gastrointestinal problems.
Meta-analysis, systematic review						
McCormick et al., 2008 [50]	DKD, *N* = 476	Systematic review (10 RCTs searched as of March 2006)	7: 1200 mg/day 1: 600 mg/day 2: 400 mg/day Treatment duration: 2 to 12 months (median 6 months)	ACEI and/or ARB (60%) or usual care	Compared with placebo or usual care, PTX may decrease proteinuria	Four patients stopped pentoxifylline therapy because of adverse effects (most common: digestive symptoms and dizziness). In the control arms, 5 patients withdrew because of adverse effects (cough due to captopril).
Shan et al., 2012 [51]	Type 1 and/or type 2 DKD, at CKD stages 3 to 4 (micro- or macro-albuminuria) *N* = 991	Cochrane systematic review (17 studies with 16 of them being RCTs, searched as of July 2009)	16: 400–1200 mg/day 1: 100 mg/day Treatment duration: 21 days to 12 months	Routine treatment plus ACEI or ARB (18%)	Evidence to support the use of PTX in reducing albuminuria & proteinuria was insufficient	Adverse effects associated with PTX were reported in nine included studies. The most common adverse effects reported were headache, dizziness, nausea and dyspepsia of mild degree.
Tian et al., 2015 [52]	Type 2 DKD, *N* = 587	Meta-analysis (8 RCTs searched as of December 2014)	5: 1200 mg/day 1: 600 mg/day 2: 400 mg/day Treatment duration: 21 days to 2 years (median 5 months)	ACEI and/or ARB	Add-on PTX to RAS blockade additively reduced proteinuria, albuminuria and urinary TNF-α. The benefits occurred independently from the decrease in BP or improvement in glycemic control.	The most frequent adverse effects in the PTX groups were transient digestive symptoms (9.4%) and dizziness (2.3%), only six participants withdrew due to intractable nausea and vomiting.
Jiang et al. 2016 [53]	CKD of various etiology, *N* = 613	Systematic review & meta-analysis (12 studies as of January 2015) - 9 RCTs: DKD 1 crossover: membranous GN 2 non-RCTs: mixed diabetic & non-diabetic kidney disease	The dose of PTX ranged from 400 to 1200 mg/day Treatment duration: 2 to 24 months	ACEI and/or ARB (one-third)	PTX decreased proteinuria compared to placebo or no-treatment groups, but the decrease was not significant compared to captopril treatment	In the pooled analysis, there was no significant difference in the risk of any adverse events between the PTX and control arms.

*BP* blood pressure, *CRP* C-reactive protein, *DKD* diabetic kidney disease, *RCT* randomized controlled trial, *UPCR* urinary protein-creatinine ratio

Later investigators consistently found that add-on PTX on top of RAS blockade led to a greater reduction of proteinuria in patients with type 2 DKD across CKD stages 1 to 3, and the benefit of which was independent of metabolic or blood pressure control [43, 44]. Of note, PTX’s anti-proteinuric activity could still be evident at a dose of 400 mg daily on top of RAS blockade over 6 months [44]. Roozbeh et al. [45] observed a modest decrease in systolic blood pressure associated with proteinuria reduction in patients treated with PTX and captopril, compared to captopril monotherapy. This was the only study reporting hypotensive activity of PTX. Recently, in a randomized double-blind, placebo-controlled trial which enrolled 174 patients with type 2 DKD across CKD stages 1 to 3, Han et al. [46] showed that add-on PTX at a dose of 1200 mg daily for 6 months reduced proteinuria and improved glucose control and insulin resistance, without decreasing serum TNF-α levels. The study was notable for having a high dropped-out rate in the PTX group (40.2%) and without measuring urinary TNF-α levels which, compared to serum TNF-α, would be more closely associated with the development of albuminuria [47, 48]. In a subsequent randomized, placebo-controlled trial, Shahidi et al. [49] observed no reduction of proteinuria by PTX therapy at a dose of 1200 mg daily for 6 months in 40 type 2 DKD patients with eGFR >60 mL/min/1.73 m^2^. Clearly, this study comprised patients with relatively normal renal function. They were not under uniform RAS blockade, yet receiving dietary protein restriction. These discrepancies in baseline characteristics and study designs might be linked to the lack of anti-proteinuric effects by PTX treatment.

Systematic reviews and meta-analyses have been employed to quantitatively evaluate PTX’s anti-proteinuric effects in diabetic patients with microalbuminuria or overt proteinuria. The first work was by McCormick et al. [50] which included 10 randomized clinical trials and a total of 476 patients with DKD. The search for literature spanned over a period from 1966 to March 2006. Compared with placebo or usual care, PTX treatment for a median duration of 6 months decreased proteinuria in patients with DKD. Patients with overt proteinuria, as opposed to microalbuminuria, had a more significant decrease in urinary protein excretion after PTX treatment. No differences in proteinuria reduction between PTX and ACEIs, and no significant changes in systolic or diastolic blood pressure, or GFR were observed after PTX treatment. The authors suggested that large high-quality studies are required. This report was followed by a Cochrane systematic review which analyzed 17 studies comprising 991 participants with DKD as of 2009 [51]. The review claimed insufficient evidence to recommend the use of PTX for DKD and called for rigorously designed, randomized, multicenter, large-scale studies. A subsequent meta-analysis by Tian et al. [52], who analyzed 8 studies including 587 patients as of December 2014. The authors concluded that PTX therapy may additively reduce proteinuria, albuminuria and urinary TNF-α in DKD patients under RAS blockade. Of note, this beneficial effect was independent from the decrease in blood pressure or improvement in glycemic control. Likewise, Jiang et al. [53] analyzed 12 studies comprising 613 patients with CKD of various etiologies including DKD as of January 2015, and summarized that PTX decreased proteinuria compared to placebo or no-treatment groups, and the decrease was not significant compared to captopril treatment. Thus, 3 out of 4 (75%) meta-analyses support the efficacy of PTX on reducing diabetic proteinuria.

### Efficacy of PTX on progression of CKD

The renoprotective potential of PTX has been eagerly reviewed [10, 11, 54–56] or meta-analyzed [57, 58] in recent years. Most such analyses, however, were based on clinical trials with varied study designs and treatment protocols, yielding inconclusive results and hampering the recommendation of PTX to the whole CKD population (Table 3). In fact, due to the potent renoprotective effect of RAS blockade, and the insidious nature of renal progression, it may be hard to observe extra benefits of PTX on top of RAS blockade, especially in studies with short treatment duration. For that reason, only outcome analyses with an average follow-up period of at least 12 months are discussed in the following sections.

**Table 3:** Trials evaluating the efficacy of PTX on renal progression with at least 1 year follow-up

Investigators, years [Ref.]	Patients, number	Study design	PTX dose, duration	Background RAS blockade	Main outcome findings	Safety profiles
Meta-analysis, systematic review						
Leporini et al., 2016 [57]	CKD of various etiology, *N* = 1518	Systematic review & meta-analysis (26 studies as of 2015) - 24: diabetic patients 2: non-diabetic or mixed CKD patients	15: 1200 mg/day 5: 800 mg/day 1: 600 mg/day 5: 400 mg/day Treatment duration: 21 days to 24 months.	15 RCTs: compared to placebo or standard therapy. 11 RCTs: compared to RAS blockade.	Lack of conclusive evidence proving the usefulness of this agent for improving renal outcomes in subjects with CKD	Mild gastrointestinal intolerance represented the most frequently reported adverse event. In a pooled meta-analysis of 10 RCTs (786 pts), PTX was associated with an almost 3-fold higher risk of gastrointestinal symptoms than control treatment.
Liu et al., 2017 [58]	CKD of various etiology, *N* = 705	Meta-analysis (11 RCT as of July 30, 2015) -8: diabetic patients 3: non-diabetic or mixed CKD patients	6: 1200 mg/day 1: 800 mg/day 1: 600 mg/day 1: 400/800 mg/day 2: 400 mg/day Treatment duration: 7: ≦6 months 4: 9 to 24 months.	11 RCTs: compared to ACEI and/or ARB	Combination of a RAS blockade and PTX reduces proteinuria & ameliorates eGFR decline in patients with CKD stages 3 to 5.	Six studies that included 218 participants reported adverse events, and the proportion was 39/218 (17.9%).
Randomized clinical trials						
Diskin et al., 2007 [65]	Insulin-dependent adult onset diabetic patients with proteinuria >1.5 g/day, *N* = 14	Open-label, controlled PTX plus ACEIs/ARBs (*n* = 7) ACEIs/ARBs alone (*n* = 7)	800 mg/day (CCr > 50 mL/min); 400 mg/day (CCr < 50 mL/min), 12 months	Maximum doses of ACEI plus ARB	Add-on PTX showed no significant benefit in proteinuria reduction or preservation of CCr.	No adverse reactions were recorded. Nevertheless, the rate of CCr decline >11 mL/min per year raises concern about the quality of patient care or the use of background maximum doses of ACEI and ARB.
Lin et al., 2008 [47]	CKD stages 3 or higher with proteinuria >0.5 g/gCr (DM 28%), N = 56	Randomized, open-label controlled PTX + losartan (*n* = 27) 0 dropped-out Losartan (*n* = 29) 2 dropped-out	800 mg/day (CKD stage 3); 400 mg/day (CKD stage 4), 12 months	Losartan (100 mg/day)	PTX treatment additively reduced proteinuria, which occurred in conjunction with changes in urinary TNF-α and MCP-1. eGFR remained stable in the PTX group but declined in the control group.	Two (7%) patients in the PTX group discontinued treatment. One patient withdrew due to recurrent gastric ulcer bleeding (at 6 months); another one withdrew due to newly diagnosed breast cancer (at 3 months) which was not related to the use of PTX.
Perkins et al., 2009 [63]	CKD stages 3 to 4 with proteinuria >1 g/day (DM 61%), N = 40	Randomized, double-blind, placebo controlled PTX + RAS blockade (*n* = 22) 5 dropped out Placebo + RAS blockade (*n* = 18) 2 dropped-out	800 mg/day, 12 months	ACEI and/or ARB	Rate of eGFR decline had been slowed in the PTX but not the control group. No effect on proteinuria was observed in the PTX group.	One participant in each group had bleeding complications and withdrew from the study. Seven (32%) participants in the PTX group and 5 (28%) in the placebo group were hospitalized for nonselective indications during the study period. No hospitalization was determined to be related to PTX.
Goicoechea et al., 2012 [64]	Type 2 diabetes with persistent proteinuria >150 mg/day; CKD stages 3 or higher, *N* = 91	Randomized, double-blind, controlled PTX (*n* = 46) 12 dropped-out Control (*n* = 45) 9 dropped-out (7 lost to follow-up)	800 mg/day (400 mg twice daily), 12 months	Usual therapy including ACEI and/or ARB (PTX: 80% Control: 82%)	PTX did not decrease proteinuria; eGFR declined in the usual therapy but not in the PTX group. Reduction of serum TNF-α, fibrinogen and hsCRP in the PTX group, although the control group also showed reduction of serum TNF-α levels.	Eight (17%) patients receiving PTX withdrew from the study because of gastrointestinal symptoms. Incomplete follow-up in both arms, particularly in the control group (16%).
Navarro-González et al., 2015 [48]	DKD at CKD stages 3 to 4, *N* = 169	Randomized, open-label controlled PTX + ARB (*n* = 82) 4 dropped-out ARB (*n* = 87) 5 dropped-out	1200 mg/day, 24 months	ARB	PTX group showed greater reduction of albuminuria and lower decrease in eGFR. Reduction of urinary TNF-α in the PTX group.	The most frequent adverse effects in patients treated with PTX were gastrointestinal symptoms (abdominal discomfort, flatus, dyspepsia, nausea, and vomiting), which were significantly more frequent than in the control group (21.9% versus 10.3%). In most cases these symptoms were self-limited and disappeared during the first month. Only one case in the PTX group discontinued treatment due to the adverse effect. Cardiovascular and cerebrovascular events and the number of hospitalizations did not differ between groups.
Observational cohort study						
Investigators, years [Ref.]	Patients, number	Study design	PTX dose, duration	Background RAS blockade	Main outcome findings	Safety profiles
Chen et al., 2014 [60]	CKD stages 3B-5 before ESRD, *N* = 661	PTX nonusers (*n* = 242, DM 44.6%) PTX users (*n* = 419, DM 54.2%)	Most patients (stages 4–5) received a PTX dose of 400 mg/day; patients in stage 3B received 800 mg/day. Median follow-up period was 2.25 years	ACEI and/or ARB	In the advanced stages of CKD, patients treated with a combination of PTX and ACEI or ARB had a better renal outcome than those treated with ACEI or ARB alone. Renoprotective effect was more prominent in patients with higher proteinuria (> 1 g/gCr).	Not available
Wu et al., 2015 [61]	CKD stage 5 (serum Cr > 6 mg/dL) plus ESA, not treated with dialysis during 6 months before and 3 months after the first prescription of ESA. N = 14,732	PTX nonusers (n = 7366, DM 39.5%) PTX users (*n* = 7366, DM 39.6%)	One-fifth to one DDD of PTX is sufficient for renoprotection. The median time from the first prescription of ESA to the initiation of dialysis and death was 1.05 years and 3.61 years, respectively.	ACEI and/or ARB (64.8%)	PTX nonusers showed an increased risk of ESRD; PTX users were protective from ESRD, compared to the nonusers who received RAS blockade monotherapy.	Not available
Kuo et al., 2015 [62]	CKD stage 5 (serum Cr > 6 mg/dL) plus prescription of ACEI or ARB within 90 days after ESA use *N* = 8742	PTX nonusers (*n* = 6354, DM 59.2%) PTX users (*n* = 2118, DM 58.9%)	No specific PTX doses or dose ranges were mentioned. Mean follow up was 11.4 months in PTX users and 12.1 months in non-users	ACEI and/or ARB	PTX exhibited a protective effect in reducing the risk for the composite outcome of long-term dialysis or death.	Not available

*CCr* creatinine clearance, *Cr* creatinine, *DDD* defined daily dose, *DM* diabetes mellitus, *ESA* erythropoiesis-stimulating agent, *ESRD* end-stage renal disease

Lin et al. [47] first reported 56 patients (72% being non-diabetic) with CKD stages 3 to 4 and urinary protein excretion >0.5 g/gCr in an open-label, randomized controlled trial. These patients had received ARB (losartan 100 mg daily) for at least 6 months at entry, and were allocated to receive either ARB or add-on PTX (400 mg once or twice daily depending on eGFR levels) to ARB. At 1 year, the add-on group displayed a lower proteinuria than the ARB group. Further analysis revealed a significant decrease of eGFR in the ARB but not the add-on PTX group at 12 months. Mechanistically, add-on PTX therapy reduced changes in urinary TNF-α and MCP-1 as compared to the ARB group. The investigators then continued the follow-up of the add-on group and added PTX to the ARB group after 1 year. The results showed that PTX treatment not only persistently decreased proteinuria, but also reproduced this benefit of add-on PTX in the ARB group over an additional follow-up of 6 months. The use of add-on PTX in this trial was well-tolerated for patients over a follow-up period of 18 months. This was the first demonstration that PTX could reduce proteinuria on top of ARB in patients with stages 3 to 4 CKD.

Then, Navarro-González et al. [48] reported the PREDIAN trial, the largest open-label, randomized controlled study to date, which comprised 169 type 2 diabetics at CKD stages 3 to 4 with albuminuria >30 mg/day under maximal RAS blockade. After 24 months of treatment, a higher reduction of albuminuria and a lower decrease in the eGFR were observed in the PTX group. The authors concluded that add-on PTX (1200 mg daily) to RAS blockade in type 2 diabetics led to a smaller decrease in eGFR and a greater reduction of residual albuminuria at 2 years. These benefits were associated with decreased urinary TNF-α in the PTX group but not in the control. Despite promising results, He & Cooper [59] pointed out several limitations in this study and suggested that a large-scale, randomized, double-blind, adequately powered, placebo-controlled, multicenter trial be undertaken to provide further evidence of the potential renoprotective effects of PTX in a real-world setting.

Most prior studies had been conducted in patients with CKD across stages 1 to 4 and used proteinuria or eGFR decline rate as surrogate outcomes. Whether PTX may exhibit efficacy on hard renal endpoints such as ESRD or death in more advanced CKD remains relatively unexplored. Recently, Chen et al. [60] analyzed 609 patients with CKD stage 3B to 5 before ESRD in a single-center observational study and found that add-on PTX provided nephroprotection in the subset of patients with high proteinuria (≧1 g/gCr). This suggests that proteinuria may be a predictor of response to PTX at the individual patient level. Then, by analyzing a nationwide administrative dataset, Wu et al. [61] identified two propensity score-matched cohorts, PTX users and nonusers, each consisting of 7366 patients, from an original population of 23,233 individuals diagnosed as advanced CKD with a serum Cr > 6 mg/dL and not treated with dialysis during 6 months before and 3 months after the first prescription of erythropoietin-stimulating agent. The authors found that the PTX users were protective from ESRD, compared to the nonusers who received RAS blockade monotherapy. Importantly, this study showed that PTX as low as 200 mg daily was sufficient to reduce the risk of new-onset ESRD in patients with advanced CKD, who often could not tolerate the use of RAS blockade. Likewise, by analyzing the same dataset of advanced CKD, Kuo et al. [62] reported that addition of PTX to background RAS blockade resulted in reduction of the risk for the composite outcome of long-term dialysis or death. Of note, the authors found no renal benefits by combining PTX with dual RAS blockade therapy compared with PTX and ARB monotherapy. Together, these cohort studies, albeit observational, provide first evidence that PTX can be an efficacious agent in reducing the risk of ESRD even in patients with late stage CKD.

Not all studies have shown concomitant anti-proteinuric and renoprotective effects by adding PTX to RAS blockade. In a double-blind, randomized, placebo-controlled design, Perkins et al. [63] examined add-on PTX (800 mg daily) to RAS blockade in 40 patients (61% being diabetic) with CKD stages 3 to 4 exhibiting proteinuria >1 g/day. At 1 year, the mean eGFR decrease was significantly less in the PTX group than the placebo group. And, for PTX-treated participants, the mean eGFR decrease during treatment was slower compared with the year before study enrollment. However, the authors did not observe PTX decreased proteinuria in comparison with the control group. They speculated that PTX might exert a greater action on tubulointerstitial injury than on glomerular filtration, and proposed that proteinuria may not always serve as an optimal surrogate outcome in studies evaluating the impact of PTX on kidney function. This study comprised a greater percentage of African Americans and patients with diabetes in the PTX group, and displayed an unusually high rates of hospitalization (28–32%) and dropped-out (17.5%), which could underestimate the anti-proteinuric potential of PTX. Later, in another open-label, randomized controlled trial, Goicoechea et al. [64] reported PTX therapy at a dose of 800 mg daily for 1 year stabilized renal function while decreasing serum inflammatory markers (TNF-α, fibrinogen and high sensitivity CRP) in 91 patients with CKD stage 3 or higher. The study, which also did not find anti-proteinuric effect of PTX in comparison to control, was limited by a high dropped-out rate (17%) in the PTX group, and incomplete follow-up in both arms, particularly the control (16%). Finally, Diskin et al. [65] reported 14 adult-onset, insulin-dependent diabetic patients with nephrotic proteinuria in an open-label, controlled trial. At 1 year, the authors did not find additive anti-proteinuric or renoprotective effects of PTX at a dose of 400–800 mg daily on background ACEIs plus ARBs. These unexpected results could be related to the nature of the study which was small and non-randomized, and the rate of creatinine clearance decline was abnormally high (>11 mL/min per year), thus raising questions about the overall quality of the trial, and the safety of dual RAS blockade [66, 67].

### Possible mechanisms underlying PTX’s renal effects

**Fig. 1:**
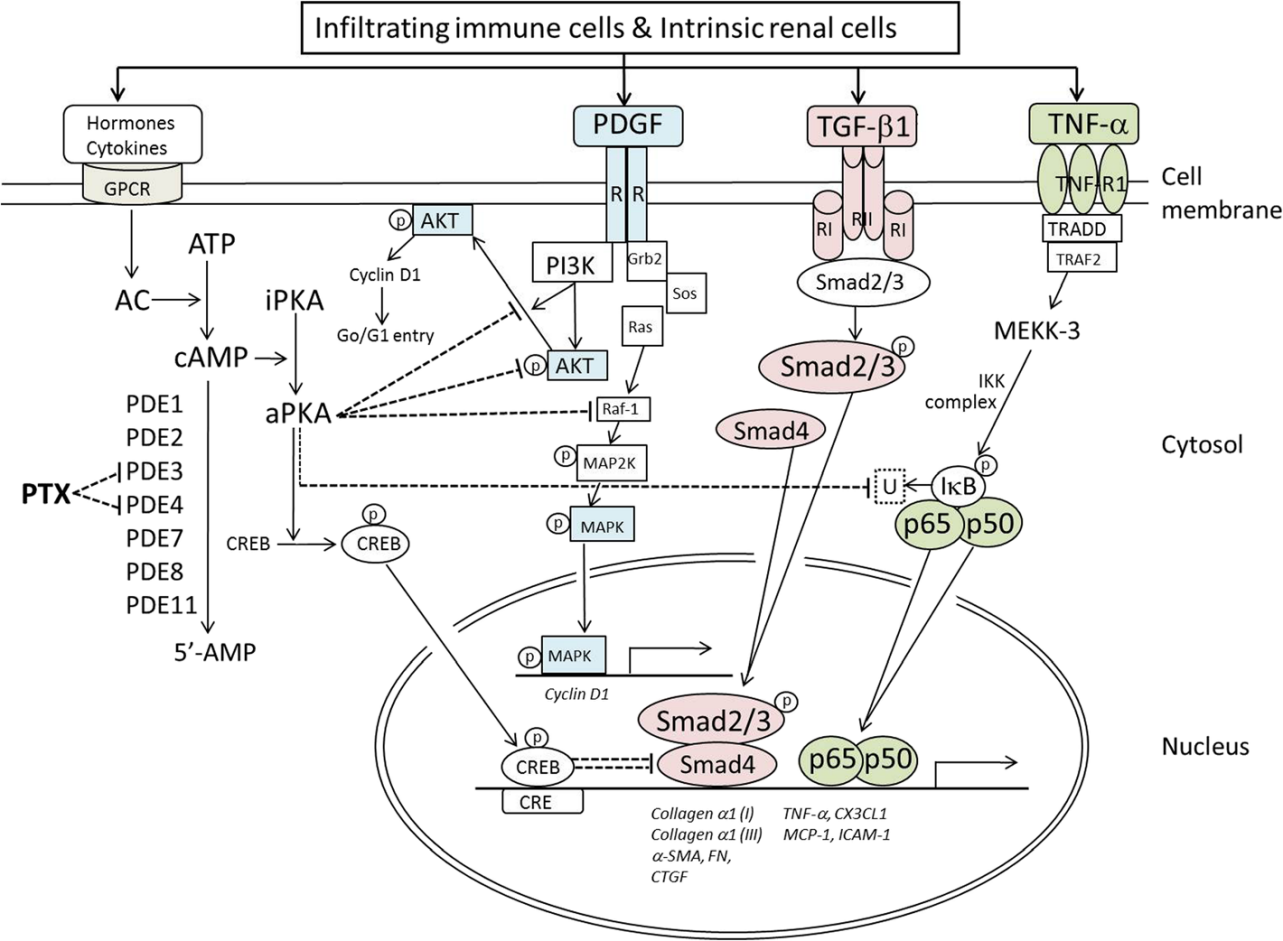
Possible mechanisms mediating PTX’s renal effects. AC, adenylate cyclase; aPKA, active protein kinase A; α-SMA, α-smooth muscle actin; ATP, adenosine triphosphate; cAMP, cyclic adenosine-3,5-monophosphate; CRE, cAMP response element; CREB, cAMP-response element binding protein; CTGF, connective tissue growth factor; CX3CL1, fractalkine; FN, fibronectin; GPCP, G-protein-coupled receptor; Grb2, growth factor receptor-bound protein 2; ICAM-1, intercellular adhesion molecule-1; IκB, inhibitory protein of NF-κB (p65/p50 heterodimer); IKK, IκB kinase; iPKA, inactive protein kinase A; MAPK, mitogen activated protein kinase; MCP-1, monocyte chemoattractant protein-1; P, phosphorylation; PDE, phosphodiesterase; PDGF, platelet-derived growth factor; PI3K, phosphatidylinositol 3-kinase; Sos, son of sevenless; TGF-β1, transforming growth factor-β1; TNF-α, tumor necrosis factor-α; PTX, pentoxifylline; TRADD, TNFR1-associated death domain protein; TRAF2, TNF receptor-associated factor 2; U, ubiquitination. Dash lines denote inhibitory pathways initiated by PTX from the leftmost side

Modulation of intracellular cyclic nucleotides by targeting PDE activity can be a novel therapeutic strategy for fibrotic kidney disease [68–70]. Currently, there are 11 PDE families with >60 isoforms which control the degradation of cyclic adenosine-3,5-monophosphate (cAMP) and cyclic guanosine-3,5-monophosphate (cGMP) within all mammalian cells [71–74]. Figure 1 illustrates the mechanisms underlying PTX’s renoprotective activities. Initially, PTX suppresses distinct cAMP PDE isozymes and elevates intracellular cAMP [75]. This results in activation of protein kinase A (PKA) activity which leads to phosphorylation of downstream effectors followed by inhibition of signaling pathways involved in proteinuria and renal fibrosis [41, 76–78]. Along this line of thought, inhibition of type 3 and type 4 PDE isozymes has been shown to suppress mitogenesis of mesangial cells and development of mesangial proliferative glomerulonephritis in rats [79, 80]. Our in vitro studies have shown that PTX acts through PKA-dependent pathway to inhibit type 3 and/or type 4 PDE isozymes, leading to elevated cAMP but not cGMP levels [76, 77, 81]. In vivo, PTX attenuates proteinuria and renal pathologies in several non-diabetic kidney disease models via modulation of signaling pathways or components triggered by cytokines (TNF-α, nuclear factor-κB, intercellular adhesion molecule-1, MCP-1 and CX3CL1/fractalkine), mitogens (platelet-derived growth factor, mitogen-activated protein kinase, phosphatidylinositol 3-kinase, Akt/protein kinase B and cyclin D1) and fibrogenic molecules (transforming growth factor-β, Smad3/4, connective tissue growth factor, collagen types 1 & 3, fibronectin and α-smooth muscle actin) [37, 78, 82–84]. In DKD models, PTX ameliorates sodium retention and renal hypertrophy together with reduced renal TNF production [85], and decrease albuminuria along with reduction of renal TNF-α, interleukin (IL)-1 and IL-6 [86]. In a subsequent report, PTX exhibits its anti-inflammatory and antioxidant activities via decreasing the expression of TNF-α and IL-6 in alloxan-induced diabetes [87]. These data indicate that PTX’s renal effects may be mediated via attenuation of proinflammatory cytokine cascades, irrespective of diabetes status.

It has been reported that TNF-α downregulate nephrin expression and cAMP-elevating agents enhance nephrin level in cultured podocytes [88]. More recently, in a mesangial proliferative glomerulonephritic model, Chen et al. [89] demonstrate that PTX can attenuate proteinuria and nephrinuria in conjunction with downregulation of the p-nuclear factor-κB and p-Smad2/3 signaling pathways, and restoration of the decreased expression for the podocyte glomerular filtration barrier, including Wilms’ tumor 1, nephrin, synaptopodin and podocin*.* These seemingly non-specific actions of PTX may turn out to be an advantage and form the basis of developing new drugs with pleiotropic activities beyond RAS blockade. Recently, a novel small molecule drug, CTP-499, which mimics the primary metabolites of PTX has demonstrated its safety and tolerability in a phase 1b randomized, double-blind, placebo controlled clinical trial in patients with CKD [90].

Apart from blocking the PDE activity, PTX can modulate other effectors or signaling pathways depending on the experimental settings. In vitro, PTX suppresses TNF-α production via PKA-independent pathway in endotoxin-activated mononuclear cells [91], and inhibits tumor growth and angiogenesis by targeting Janus kinase-signal transducer and activator of transcription signaling [92]. In vivo, PTX attenuates endotoxin-induced acute lung injury via adenosine receptor A_2A_-dependent cascade [93], and ameliorates alloxan-induced diabetes by reduction of inducible nitric oxide synthase system [87]. Whether these TNF-α-independent mechanisms play a role in mediating PTX’s effects still awaits further studies.

### Safety profile of PTX use in patients with kidney disease

The most common adverse effects of PTX are gastrointestinal symptoms and dizziness [15, 52, 94]. Presumably, these effects of PTX may occur more frequently in patients whose dosages are not tailored by degrees of renal dysfunction. For instances, Renke et al. [25] observed a relatively high incidence of adverse effects, i.e., nausea, dyspepsia and diarrhea in 5 patients (23%) during the study period. They considered this could be ascribed to no reduction of PTX doses in patients with moderate renal dysfunction, which resulted in accumulation of PTX metabolites and gastrointestinal intolerance. Also in the PREDIAN trial [48], the incidence of abdominal discomfort, flatus, dyspepsia, nausea, and vomiting in patients treated with PTX was significantly higher than in the control group (21.9% versus 10.3%). That said, these digestive symptoms were generally self-limited and usually disappeared when used continuously with proper dose adjustment beyond the first month. During multidose pharmacokinetic studies, accumulation of active metabolites IV and V has been documented in patients with renal impairment. For that reason, dose reduction to 400 mg twice daily and 200–400 mg daily is advised for patients with creatinine clearances between 30 and 80, and <30 mL/min, respectively [95, 96].

There exists concern that PTX might exert a negative impact on glycemic control in patients with diabetes. Previous studies have shown that PDE3,4 inhibitors enhance both glycogenolysis and gluconeogenesis in hepatocytes isolated from fasted rats [97], and the blood glucose level increases following the administration of cilostazol, a PDE3 inhibitor, in three patients with type 2 diabetes [98]. More recently, however, PDE4 inhibitors have been shown to improve glucose homeostasis in diabetic mice [99] and human patients [100], likely via enhancing the secretion of cAMP-mediated glucagon-like peptide 1 [99]. Further, of all the literatures reviewed (Tables 1,2,3), there was no report that PTX increased blood glucose in diabetic patients with overt proteinuria or CKD of various stages. In a meta-analysis of 499 patients with type 2 diabetic nephropathy from 6 clinical trials, Tian et al. [52] observed no significant change of HbA1c in the PTX group compared with that of the control. On top of that, Han et al. [46] reported that add-on PTX reduced proteinuria in conjunction with improved glucose control and insulin resistance in patients with type 2 diabetes.

## Conclusions

PTX is a nonselective PDE inhibitor that exhibits anti-inflammatory, anti-proliferative and anti-fibrotic actions both in vitro and in vivo. Current evidence suggests that PTX, used either alone or in combination with RAS blockade, may have an adjunct role of renoprotection for CKD. Nevertheless, most published literatures were limited by small sample size, short observation period and imperfect methodology using surrogate outcomes (proteinuria, eGFR decline). There is need for more well-designed studies with longer duration of follow-up aiming at hard renal endpoints (ESRD, doubling of serum creatinine). Because PTX is an old drug that lacks financial sponsorship, future researches examining the efficacy PTX on a background of RAS blockade would better be conducted on patients with more severe CKD, e.g., stage 3B or higher, so that hard outcomes can be evaluated using a minimum number of subjects within an acceptable period of time.

## Abbreviations

ACEI: Angiotensin converting enzyme inhibitor
ARB: Angiotensin receptor blockers
cAMP: Cyclic adenosine-3,5-monophosphate
cGMP: Cyclic guanosine-3,5-monophosphate
CKD: Chronic kidney disease
CRP: C-reactive protein
DKD: Diabetic kidney disease
eGFR: Estimated glomerular filtration rate
ESRD: End-stage renal disease
IL: Interleukin
MCP: Monocyte chemoattractant protein
PDE: Phosphodiesterase
PKA: Protein kinase A
PTX: Pentoxifylline
RAS: Renin-angiotensin system
TNF: Tumor necrosis factor
